# Research progress of cuproptosis, ferroptosis, apoptosis, and autophagy in knee osteoarthritis

**DOI:** 10.3389/fcell.2026.1855681

**Published:** 2026-07-01

**Authors:** He Shang, Tao Ma, Yuan Wei, Jinpeng Liang, Yi Wang, Jichen Liu, Tianxiang Yang, Xueqi Liu, Yinbin Wang, Xing He, Yumei Ding, Jun Li, Biao Ma, Yaxing Ma, Ruoyu Wang, Desheng Chen

**Affiliations:** 1 Third Clinical Medical College of Ningxia Medical University, People’s Hospital of Ningxia Hui Autonomous Region, Yinchuan, China; 2 Department of Joint Surgery, Ningxia Hui Autonomous Region People’s Hospital (Affiliated Hospital of Ningxia Medical University), Yinchuan, China; 3 Department of Pediatrics, Affiliated Hospital of Xuzhou Medical University, Xuzhou, China

**Keywords:** apoptosis, autophagy, chondrocyte, crosstalk, cuproptosis, disease-modifying osteoarthritis drugs, ferroptosis, knee osteoarthritis

## Abstract

Knee osteoarthritis (KOA) is a highly prevalent chronic degenerative joint disorder characterized by progressive articular cartilage degeneration, chondrocyte death, and extracellular matrix degradation. Accumulating evidence indicates that dysregulated programmed cell death (PCD) pathways—including cuproptosis, ferroptosis, and apoptosis—along with autophagic dysfunction play pivotal roles in KOA pathogenesis. Cuproptosis is a recently identified copper-dependent cell death modality triggered by mitochondrial copper overload, leading to aggregation of lipoylated TCA cycle enzymes, destabilization of iron-sulfur clusters, and proteotoxic stress. Ferroptosis is an iron-dependent regulated cell death pathway driven by lethal lipid peroxidation and GPX4 inactivation. Apoptosis, the classical caspase-dependent PCD, contributes to chondrocyte loss through extrinsic and intrinsic pathways. Autophagy exhibits a context-dependent dual role, serving cytoprotective functions under physiological conditions yet becoming detrimental when dysregulated. Importantly, these pathways exhibit marked stage-dependent and cell-type-specific characteristics during KOA progression: their activity profiles differ substantially between early, middle, and advanced disease, and among chondrocytes, synovial fibroblasts, macrophages, and subchondral bone cells. These four processes engage in intricate crosstalk through shared molecular nodes, including ROS, GSH metabolism, mitochondrial dynamics, and mTOR/NF-κB signaling. This review provides a comprehensive synthesis of the core molecular mechanisms, critically evaluates the stage-dependent and cell-type-specific roles in KOA pathogenesis, and systematically maps the crosstalk network with causal relationships. We also discuss current therapeutic strategies (metal chelators, natural compounds, nanoparticle systems) with detailed assessment of their mechanisms, limitations, and translational bottlenecks, and propose future research directions for developing multi-target disease-modifying osteoarthritis drugs (DMOADs). Specific clinical implications—including biomarker potential, patient stratification, and stage-guided personalized therapy—are also highlighted.

## 1 Introduction

Knee osteoarthritis (KOA) is a highly prevalent chronic degenerative joint disorder globally, with epidemiological data indicating a prevalence exceeding 30% among individuals aged over 65 years, and a persistent upward trend observed worldwide (Saengsiwaritt et al., 2023; Liu Y. et al., 2024). The core pathological hallmark of KOA involves progressive articular cartilage degeneration, characterized by diminished chondrocyte viability and functional impairment, coupled with disrupted homeostasis of the extracellular matrix—specifically reduced synthesis and accelerated degradation of type II collagen and proteoglycans (Zhang Y. et al., 2025; Sheng et al., 2024). Clinically, this manifests as persistent joint pain, morning stiffness, restricted mobility, and, in advanced stages, joint deformity and functional disability (Shen et al., 2025).

While multifactorial etiologies including aging, obesity, mechanical stress, and genetic predisposition contribute to KOA pathogenesis (Wang T. et al., 2025), accumulating evidence underscores dysregulated programmed cell death (PCD) pathways and autophagic dysfunction as pivotal drivers of chondrocyte injury and cartilage deterioration (Huang et al., 2025; Liu et al., 2025). Apoptosis, the earliest identified PCD mechanism, is well-documented in OA cartilage degradation; however, therapeutic strategies solely targeting apoptosis have proven insufficient to halt disease progression (Zhao et al., 2025; Liu et al., 2023). Autophagy functions as a double-edged sword in KOA: it serves as a cytoprotective mechanism under physiological conditions by clearing damaged organelles and macromolecules to maintain chondrocyte homeostasis, yet becomes detrimental when dysregulated—either deficient or excessive—thereby exacerbating cartilage matrix loss and inflammatory responses (Mei et al., 2026). Ferroptosis, an iron-dependent regulated cell death pathway driven by lethal lipid peroxidation and closely linked to iron overload and oxidative stress, has emerged as a critical contributor to chondrocyte demise in OA (Huang et al., 2025). Concurrently, cuproptosis, a recently defined copper-dependent cell death modality triggered by copper-induced lipoylated protein aggregation and mitochondrial dysfunction, is increasingly implicated in OA pathogenesis due to dysregulated copper homeostasis affecting bone and cartilage cell function (Shen et al., 2025).

Notably, apoptosis, autophagy, ferroptosis, and cuproptosis do not operate in isolation. Substantial crosstalk exists among these pathways through shared molecular nodes, including reactive oxygen species (ROS) generation, glutathione (GSH) metabolism, mitochondrial dynamics, and inflammatory signaling cascades (Malla et al., 2025; Zheng et al., 2025). For instance, the glutathione pathway and autophagy machinery critically link ferroptosis and cuproptosis, while mitophagy modulates ferroptotic susceptibility in chondrocytes (Xie Y. et al., 2025; Jiang and Shen, 2026). Dysregulation of copper and iron homeostasis, potentially driven by inflammatory mediators, may synergistically amplify metal-mediated cell death in joint tissues (Shen et al., 2025). Bioinformatic analyses further reveal coordinated expression patterns of ferroptosis- and cuproptosis-related genes (e.g., SLC7A11, GPX4, ATP7B, GLS) correlating with cartilage degeneration, immune infiltration, and disease severity in OA (Wang J. et al., 2026; Shi et al., 2024). Therapeutic exploration is advancing toward multi-target strategies: natural compounds (e.g., quercetin) and repurposed drugs (e.g., metformin) demonstrate efficacy in attenuating ferroptosis and cuproptosis via antioxidant reinforcement, copper chelation, or pathway modulation (Huang et al., 2025); nanoparticle-based systems enabling synergistic induction of cuproptosis/ferroptosis combined with immunotherapy also show promise (Adzavon et al., 2024; Tang et al., 2025; Wu et al., 2024).

Elucidating the intricate interplay among these cell death modalities and autophagy provides a robust theoretical foundation for identifying novel disease-modifying targets in KOA. Future research should prioritize mechanistic dissection of pathway crosstalk in chondrocytes and translation of multi-death-pathway-targeted interventions to address the unmet clinical need for effective KOA therapeutics (Sheng et al., 2024; Shen et al., 2025; Huang et al., 2025; Liu et al., 2025). The subsequent sections will systematically address the stage-dependent dynamics and cell-type specificity of these pathways, followed by an integrated crosstalk analysis and a critical evaluation of therapeutic strategies and translational hurdles.

## 2 Core mechanisms of cuproptosis, ferroptosis, apoptosis, and autophagy

### 2.1 Core mechanisms of cuproptosis

Cuproptosis represents a copper-dependent, regulated form of cell death distinct from apoptosis, ferroptosis, necroptosis, and other canonical pathways. Its defining molecular mechanism involves mitochondrial copper overload, wherein excess copper ions bind directly to lipoylated components of the tricarboxylic acid (TCA) cycle—particularly dihydrolipoyl transacetylase (DLAT)—triggering their aggregation, destabilization of iron–sulfur clusters, and consequent proteotoxic stress that culminates in irreversible mitochondrial dysfunction (Song X. H. et al., 2025; Hu and Jiang, 2025). This process is critically dependent on ferredoxin 1 (FDX1), a mitochondrial iron–sulfur cluster-containing protein that functions as an electron shuttle: FDX1 reduces Cu^2+^ to Cu^+^ and facilitates protein lipoylation, thereby enabling copper-mediated protein misfolding and aggregation (Hsiao et al., 2025). Depletion or inhibition of FDX1 robustly suppresses cuproptosis, confirming its indispensable role as both a molecular executor and biomarker (Ma et al., 2025; Wang B. et al., 2022).

**FIGURE 1:**
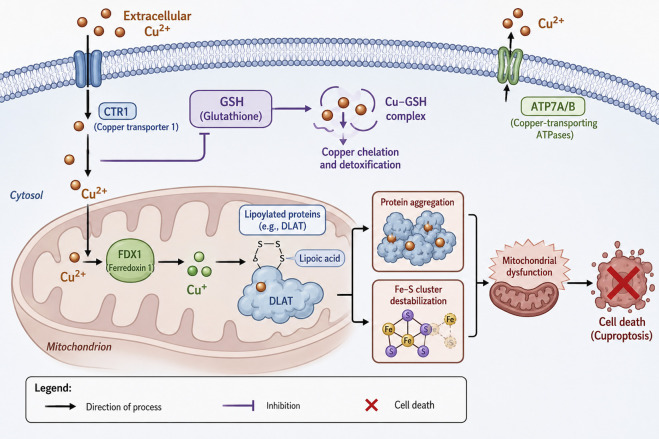
Schematic diagram of the core mechanism of cuproptosis. Extracellular Cu^2+^ enters the cell via CTR1 (copper transporter 1), then is reduced to Cu^+^ by FDX1 (ferredoxin 1) in the mitochondria. Cu^+^ binds to lipoylated proteins (e.g., DLAT, dihydrolipoyl transacetylase), causing their aggregation, iron-sulfur cluster destabilization, and mitochondrial dysfunction, leading to cell death. GSH (glutathione) chelates copper and protects against cuproptosis. ATP7A/B export copper out of the cell.

Under physiological conditions, cellular copper homeostasis is tightly regulated by influx transporters (e.g., SLC31A1/CTR1), efflux ATPases (ATP7A/ATP7B), and copper chaperones (Zhao and Qi, 2023; Xie L. et al., 2025). Disruption of this balance—via exogenous copper overload, ionophore exposure (e.g., elesclomol), or impaired copper export—precipitates cuproptosis (Lei et al., 2023; Kuang et al., 2025). Morphologically, affected cells exhibit mitochondrial shrinkage, loss of membrane potential, respiratory chain impairment, elevated ROS, and DLAT oligomerization, without classical apoptotic or lipid peroxidation features (Li H. et al., 2025). Although ROS generation occurs during cuproptosis, the primary lethal event is proteotoxic stress from aggregated lipoylated proteins rather than lipid peroxidation (Lu et al., 2023). GSH depletion—via reduced NADPH availability or G6PD inhibition—diminishes cellular copper buffering capacity and sensitizes cells to cuproptosis, highlighting the interplay between redox homeostasis and copper toxicity (Gao et al., 2026) (Figure 1).

FDX1 expression levels significantly influence tumor susceptibility to cuproptosis. In clear cell renal cell carcinoma (ccRCC) and lung adenocarcinoma (LUAD), high FDX1 correlates with better prognosis and enhanced sensitivity to copper ionophores (Zhao et al., 2026; Liu S. et al., 2024). Conversely, FDX1 downregulation in hepatocellular carcinoma (HCC) or lenvatinib-resistant cells confers resistance, while its overexpression restores cuproptotic vulnerability (Sun et al., 2024; Yang et al., 2025). Regulatory networks involving transcription factors (e.g., CEBPA), long non-coding RNAs (e.g., PVT1), and metabolic enzymes (e.g., G6PD) further modulate FDX1 activity and cuproptosis efficiency. Notably, natural compounds (e.g., quercetin, TLB) and nanomaterials can target FDX1 to either induce or inhibit cuproptosis, offering therapeutic avenues in oncology and toxicology (Wei J. et al., 2025; Zhang X. et al., 2025).

Emerging evidence also implicates cuproptosis in non-neoplastic pathologies, including cardiovascular diseases, endometriosis, and nanoparticle-induced pulmonary inflammation, underscoring its broad pathophysiological relevance (Huang et al., 2026; Wang et al., 2023a). Ongoing research focuses on elucidating structural determinants of FDX1 (e.g., residues D136/D139), developing FDX1-targeted therapeutics, and exploring synergies with existing treatments. As a redox-sensitive vulnerability in cancer and other diseases, cuproptosis represents a promising frontier for precision medicine (Wu A. et al., 2025; Cao et al., 2023a; Jiang D. et al., 2025). These mechanistic features have direct implications for KOA pathogenesis, as discussed in Section 3.1.

### 2.2 Core mechanisms of ferroptosis

Ferroptosis is a regulated, iron-dependent form of non-apoptotic cell death characterized by the lethal accumulation of phospholipid hydroperoxides, particularly on membranes rich in polyunsaturated fatty acids (PUFAs), leading to loss of plasma membrane integrity (Wei M. et al., 2025; Wang et al., 2023b). While iron is essential for physiological processes such as hemoglobin synthesis and redox reactions, dysregulation of cellular iron homeostasis—mediated by transferrin receptor 1 (TFR1) for uptake, ferritin heavy/light chains (FTH1/FTL) for storage, and ferroportin 1 (FPN1) for export—results in labile iron pool expansion. Excess free Fe^2+^ catalyzes Fenton reactions, generating hydroxyl radicals (·OH) that initiate peroxidation of membrane PUFAs (Wang et al., 2023c; Sajeev et al., 2026). This process is amplified by enzymes such as acyl-CoA synthetase long-chain family member 4 (ACSL4), which esterifies PUFAs into phospholipids susceptible to oxidation, and lipoxygenases (LOXs), which directly catalyze lipid peroxidation (Wang Z. et al., 2026; Hu et al., 2024).

The primary cellular defense against ferroptosis centers on the GSH-glutathione peroxidase 4 (GPX4) axis. GPX4 utilizes GSH to reduce phospholipid hydroperoxides into non-toxic alcohols, thereby halting membrane damage (Sheng et al., 2025; Wei et al., 2020). GSH biosynthesis depends on cystine import via system Xc^−^, a heterodimeric transporter composed of solute carrier family 7 member 11 (SLC7A11) and SLC3A2; SLC7A11 dysfunction depletes GSH, impairing GPX4 activity and accelerating ferroptosis (Chen B. et al., 2024; Cui et al., 2025; Wu B. et al., 2025). Consequently, SLC7A11 and GPX4 function as pivotal ferroptosis suppressors, whereas ACSL4 and LOXs act as key promoters.

**FIGURE 2:**
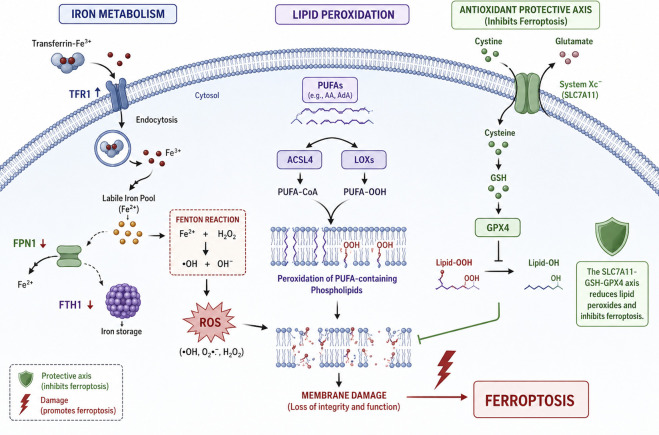
Schematic diagram of the core mechanism of ferroptosis. Iron uptake via TFR1 increases labile iron pool, while FPN1 and FTH1 are downregulated. Fe^2+^ drives Fenton reaction to produce ROS. ACSL4 and LOXs promote peroxidation of PUFAs in membrane phospholipids, leading to membrane damage. The SLC7A11-GSH-GPX4 axis reduces lipid peroxides and inhibits ferroptosis.

Beyond the canonical GSH-GPX4 pathway, multiple parallel antioxidant systems fine-tune ferroptosis sensitivity: the ferroptosis suppressor protein 1 (FSP1)-coenzyme Q10 (CoQ10) axis, the GTP cyclohydrolase-1 (GCH1)-tetrahydrobiopterin (BH4) pathway, and the dihydroorotate dehydrogenase (DHODH)-CoQH_2_ system provide GPX4-independent protection (Wang J. G. et al., 2026). Regulatory layers further modulate these effectors through transcriptional control (e.g., p53-mediated repression of SLC7A11 (Li et al., 2021), Nrf2 activation of SLC7A11/GPX4 (Li P. et al., 2024)), epigenetic mechanisms (e.g., m^6^A modification regulating TFAP2A-driven SLC7A11/GPX4 transcription (Li P. et al., 2025)), and post-translational modifications (e.g., CD38 stabilizing SLC7A11 by competing with TRIM21 (Li W. et al., 2025); Otub1 reinforcing SLC7A11/GSH/GPX4 cascades via interaction with Kindlin-2) (Dong et al., 2025). Crosstalk with mitophagy, autophagy (e.g., ferritinophagy), and non-coding RNAs (e.g., lncPVT1/miR-214-3p/GPX4 axis) further integrates ferroptosis into broader cellular stress responses (He et al., 2021; Chen et al., 2021) (Figure 2).

Dysregulated ferroptosis contributes significantly to diverse pathologies, including ischemia-reperfusion injury (cardiac, cerebral, renal) (Shan et al., 2024), neurodegenerative disorders, diabetic complications, atherosclerosis, and cancer progression or therapy resistance (Wang et al., 2024). Targeting ferroptosis regulators—such as pharmacological activation of Nrf2/SLC7A11/GPX4 or inhibition of SLC7A11 by triptolide (Liu et al., 2022)—represents a promising therapeutic strategy. Continued elucidation of context-dependent regulatory networks, including mitochondrial dynamics and metabolic reprogramming, will advance precision interventions across disease spectra. These mechanistic features are directly relevant to KOA, as discussed in Section 3.2.

### 2.3 Core mechanisms of apoptosis

Apoptosis represents a highly regulated, caspase-dependent form of PCD, characterized by distinct morphological features including cell shrinkage, chromatin condensation, nuclear fragmentation, membrane blebbing, and formation of apoptotic bodies, all occurring without eliciting inflammatory responses—thereby preserving tissue integrity and homeostasis (Mo et al., 2025; Shoshan-Barmatz et al., 2023). Two principal signaling cascades govern apoptotic execution: the extrinsic (death receptor-mediated) pathway and the intrinsic (mitochondrial) pathway.

The extrinsic pathway is initiated upon ligand binding (e.g., FasL, TNF-α) to cell surface death receptors such as Fas (CD95) or TNFR1, leading to formation of the death-inducing signaling complex (DISC), recruitment of FADD, and activation of initiator caspase-8. Active caspase-8 subsequently cleaves and activates effector caspases (e.g., caspase-3, -7), directly executing apoptosis. In certain cellular contexts, caspase-8 cleaves Bid to generate truncated Bid (tBid), which bridges to the intrinsic pathway (Song and Fan, 2018).

**FIGURE 3:**
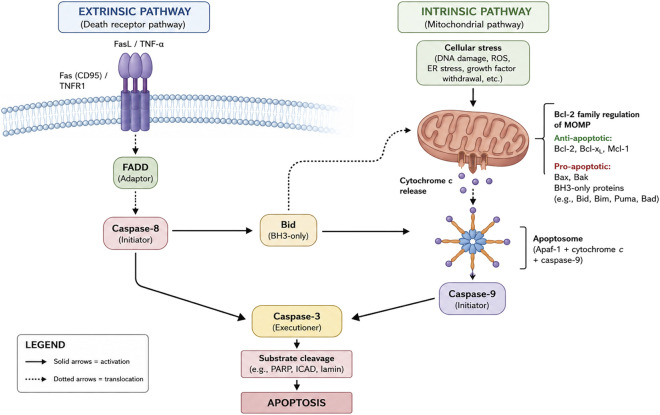
Schematic diagram of the core mechanism of apoptosis. Extrinsic pathway: death receptor (Fas/TNFR1) → FADD → caspase-8 → caspase-3. Intrinsic pathway: mitochondrial stress → cytochrome c release → apoptosome (Apaf-1 + caspase-9) → caspase-3. Bid provides cross-talk. Bcl-2 family proteins regulate MOMP.

The intrinsic pathway is triggered by intracellular stressors—including ROS, DNA damage, hypoxia, or growth factor deprivation—resulting in mitochondrial outer membrane permeabilization (MOMP), loss of mitochondrial membrane potential (ΔΨm), and cytosolic release of apoptogenic factors such as cytochrome c and apoptosis-inducing factor (AIF). Cytochrome c binds Apaf-1 and procaspase-9 to form the apoptosome, activating caspase-9, which then activates effector caspases (Yaguchi et al., 2023; Zhang et al., 2017). AIF may also mediate caspase-independent apoptosis under specific conditions (Figure 3).

Central regulators include the Bcl-2 protein family, categorized into anti-apoptotic members (Bcl-2, Bcl-xL, Mcl-1) that preserve mitochondrial integrity, and pro-apoptotic members: multi-domain effectors (Bax, Bak) that induce MOMP, and BH3-only proteins (Bid, Bim, Bad, Puma, Noxa) that either activate Bax/Bak or neutralize anti-apoptotic counterparts (Salam et al., 2018). The Bax/Bcl-2 ratio serves as a critical rheostat determining cellular fate (Liu, 2018). Caspases function as cysteine-aspartic proteases: initiators (caspase-8 for extrinsic; caspase-9 for intrinsic) activate executioners (caspase-3, -7), which cleave cellular substrates (e.g., PARP), dismantling the cell (Susanto et al., 2024; Barman et al., 2018; P et al., 2018). Additionally, cross-talk between pathways occurs via Bid cleavage and shared downstream effectors.

The tumor suppressor p53 acts as a pivotal apoptosis inducer through dual mechanisms: (1) transcriptional upregulation of pro-apoptotic genes (e.g., Bax, Puma, Noxa, Fas) and repression of anti-apoptotic genes (e.g., Bcl-2); (2) direct mitochondrial translocation where it interacts with Bcl-2 family proteins to promote MOMP (Wei et al., 2023). Dysregulation of these pathways—such as overexpression of Bcl-2/Bcl-xL or impaired p53 function—contributes to apoptosis resistance in cancer and other diseases, underscoring their therapeutic relevance (Aniogo et al., 2020). Comprehensive understanding of these molecular networks continues to inform targeted strategies for modulating cell survival in oncology and beyond (Ahmed and Tait, 2025). Section 3.3 discusses how these apoptotic mechanisms are dysregulated in KOA chondrocytes and other joint cells.

### 2.4 Core machinery of autophagy

Autophagy is a highly conserved intracellular degradation process mediated by lysosomes, responsible for eliminating damaged organelles, misfolded proteins, and metabolic waste to maintain cellular homeostasis and metabolic equilibrium (Zhu C. et al., 2024; Arias et al., 2020). Based on mechanistic distinctions, autophagy is categorized into macroautophagy, microautophagy, and chaperone-mediated autophagy. Among these, macroautophagy (hereafter referred to as autophagy) has garnered predominant attention in KOA research due to its critical role in chondrocyte survival and cartilage integrity (Lee et al., 2024; Wang H. et al., 2025; Kuwahara et al., 2022).

**FIGURE 4:**
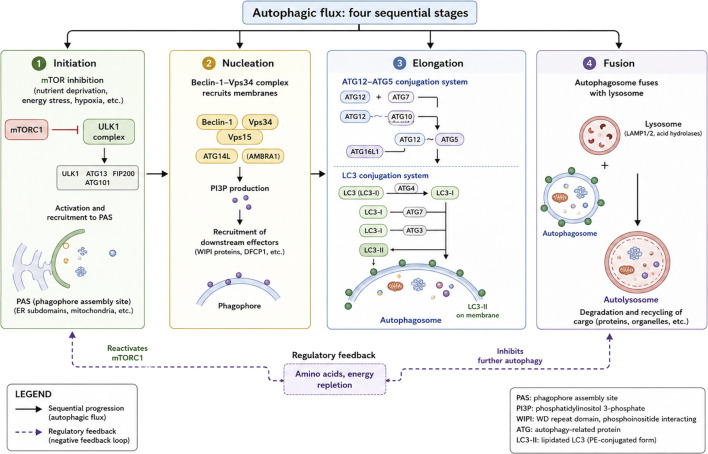
Schematic diagram of the core machinery of autophagy. Four stages of autophagic flux: Initiation (mTOR inhibition → ULK1 complex activation), Nucleation (Beclin-1-Vps34 complex), Elongation (ATG5-ATG12 and LC3-II systems), Fusion (autophagosome + lysosome → autolysosome). Key molecules are shown.

The canonical autophagy cascade comprises four tightly regulated phases: initiation, nucleation, elongation, and fusion. Initiation is primarily governed by the mechanistic target of rapamycin (mTOR) signaling pathway; under nutrient-rich conditions, active mTOR suppresses the ULK1 complex, whereas cellular stressors (e.g., nutrient deprivation, oxidative stress) inhibit mTOR, thereby activating autophagy-related genes (ATGs) and triggering phagophore formation (Huang et al., 2023; Wan et al., 2019). Nucleation involves the Beclin-1–Vps34 (PI3KC3) complex, essential for generating the isolation membrane. During elongation, ATG5–ATG12 and LC3-II conjugation systems facilitate expansion of the double-membrane autophagosome, which sequesters cytoplasmic cargo. Finally, fusion with lysosomes forms autolysosomes, where lysosomal hydrolases degrade contents, and resultant metabolites (e.g., amino acids, fatty acids) are recycled for biosynthesis and energy production (Mei et al., 2026; Jeon and Im, 2017) (Figure 4).

Key regulatory pathways include:

1. mTOR pathway: Serves as the central inhibitory node; its suppression (e.g., by rapamycin, polydatin, or geniposide) activates autophagy and exerts chondroprotective effects in KOA models (Ye et al., 2024).
2. AMPK pathway: Activated under low-energy states, AMPK phosphorylates and inhibits mTOR while directly promoting ULK1 activity, thereby enhancing autophagy. Geniposide and artesunate (ART) alleviate OA progression via AMPK/mTOR-mediated autophagy induction (Huang et al., 2023).
3. PI3K/AKT/mTOR axis: Frequently dysregulated in OA; inhibition of this pathway (e.g., by Tuina therapy, saikosaponin D, or QHJD formula) restores autophagy, reduces chondrocyte apoptosis, and suppresses inflammation (Chen and Zhang, 2024; Zhao et al., 2023).

Additional modulators: ROS, endoplasmic reticulum stress, microRNAs (e.g., miR-31, miR-128a, miR-20), and non-coding RNAs fine-tune autophagy in chondrocytes. For instance, miR-20 targets ATG10 via PI3K/AKT/mTOR to suppress autophagy, while DPSC-derived exosomes deliver miR-31 to inhibit mTOR and promote autophagy (Zhao et al., 2023; An et al., 2023).

Autophagy exhibits a context-dependent dual role in KOA pathogenesis. Physiological autophagy maintains chondrocyte homeostasis by clearing damaged mitochondria (mitophagy), aggregated proteins, and senescent components, thereby counteracting oxidative stress, inflammation, and extracellular matrix degradation (Lv X. et al., 2022). However, age-related or pathological autophagy impairment leads to accumulation of dysfunctional organelles and macromolecules, accelerating chondrocyte senescence, apoptosis, and cartilage degeneration (D'Adamo et al., 2017). Conversely, excessive or dysregulated autophagy may contribute to autophagic cell death under severe stress, highlighting the necessity of balanced autophagic flux (Andrei et al., 2024). Notably, mitochondrial autophagy (mitophagy) is particularly crucial in KOA, as mitochondrial dysfunction drives OA progression; bioinformatic analyses have identified mitophagy-related genes as potential biomarkers and therapeutic targets.

Emerging evidence supports autophagy modulation as a promising disease-modifying strategy for KOA. Natural compounds (e.g., resveratrol, curcumin, polydatin), traditional therapies (e.g., Tuina), and exosome-based interventions activate protective autophagy via mTOR-dependent or AMPK-mediated pathways, attenuating pain, synovitis, and cartilage damage in preclinical models (Cheema et al., 2025). Furthermore, autophagy-related proteins (e.g., LC3, Beclin-1, p62) in serum or synovial fluid may serve as diagnostic or prognostic biomarkers. Future research should focus on stage-specific autophagy regulation, cell-type-specific delivery systems, and clinical translation of autophagy-targeted therapeutics to achieve precision management of KOA. Section 3.4 discusses the stage-dependent dual role of autophagy in KOA in detail.

Comparison of core features of cuproptosis, ferroptosis, apoptosis, and autophagy (Table 1).

**TABLE 1:** Comparison of core features among cuproptosis, ferroptosis, apoptosis, and autophagy.

Feature	Cuproptosis	Ferroptosis	Apoptosis	Autophagy
Primary trigger	Copper overload	Iron overload, GPX4 inhibition	Death receptor activation, mitochondrial damage	Nutrient deprivation, oxidative stress
Key regulators	FDX1, DLAT, ATP7A/B	GPX4, SLC7A11, ACSL4	Caspases, Bcl-2 family	mTOR, Beclin-1, LC3
Key metabolic pathway	Lipoylated TCA enzyme aggregation	Lipid peroxidation, GSH depletion	Mitochondrial permeabilization	Lysosomal degradation
Morphological features	Mitochondrial shrinkage, cristae loss	Mitochondrial shrinkage, increased membrane density	Cell shrinkage, nuclear condensation	Autophagosome formation
Inflammatory response	Pro-inflammatory (DAMPs release)	Pro-inflammatory (lipid mediators)	Non-inflammatory	Bidirectional regulation
Role in KOA	Promotes chondrocyte death, ECM degradation	Promotes chondrocyte death, synovitis	Promotes chondrocyte loss	Protective in early stage, dysregulated in advanced stage

## 3 Roles of cuproptosis, ferroptosis, apoptosis, and autophagy in the pathogenesis of KOA

### 3.1 Cuproptosis in KOA

#### 3.1.1 Dysregulation of cuproptosis in KOA

Recent research has substantiated that aberrant activation of cuproptosis plays a pivotal role in KOA pathogenesis. However, the dysregulation pattern is highly stage-dependent and cell-type-specific. Clinical evidence demonstrates significantly elevated copper concentrations in articular cartilage and synovial fluid of KOA patients compared to healthy controls, with copper levels positively correlating with histological severity of cartilage degeneration (Zhou H. et al., 2025). In early KOA, mild to moderate copper elevation and moderate FDX1 upregulation are observed, but chondrocytes retain partial compensatory GSH levels. In advanced KOA, marked copper overload, high FDX1/DLAT expression, and severe GSH depletion are typical. Chondrocytes from KOA tissues exhibit hallmark features of cuproptosis, including mitochondrial swelling and loss of cristae structure.

Transcriptomic analyses reveal dysregulated expression of cuproptosis-related genes (CRGs) in KOA cartilage. Specifically, upregulation of cuproptosis-promoting factors (FDX1, LIAS, DLAT) and downregulation of protective regulators (GSS, ATP7A) heighten chondrocyte susceptibility to copper-induced death (Zhou F. et al., 2025). Cell-type specifically: in synovial fibroblasts, elevated expression of the copper importer SLC31A1 (CTR1) enhances copper uptake, inducing cuproptosis and amplifying secretion of pro-inflammatory cytokines (IL-6, TNF-α), thereby exacerbating synovitis and secondary cartilage damage (Han et al., 2024). In subchondral bone cells, cuproptosis-related gene expression is less characterized but emerging evidence suggests a potential role in abnormal bone remodeling. This crosstalk between cuproptosis and immune microenvironment dysregulation underscores copper’s dual role in joint homeostasis and pathology (Jiang Z. et al., 2025).

Preclinical validation in murine KOA models (e.g., destabilization of medial meniscus) confirms elevated intra-articular copper levels, increased FDX1 expression, and accelerated cartilage degradation with concomitant cuproptotic chondrocyte loss (Wang et al., 2023d). Notably, intervention with the copper chelator tetrathiomolybdate (TTM) significantly attenuates chondrocyte cuproptosis, suppresses synovial inflammation, and preserves cartilage integrity, highlighting the therapeutic potential of copper-targeting strategies.

#### 3.1.2 Mechanisms of cuproptosis regulation in KOA

In chondrocyte injury, dysregulated copper homeostasis leads to intracellular copper accumulation, which triggers cuproptosis via dual mechanisms: (i) direct binding to lipoylated TCA cycle enzymes (e.g., DLAT, PDHA1), inducing protein aggregation, mitochondrial respiratory dysfunction, and energy metabolism collapse; (ii) catalyzing ROS generation, promoting lipid peroxidation and membrane damage, while upregulating pro-inflammatory cytokines (TNF-α, IL-1β, IL-6) that exacerbate cellular stress. Concurrently, diminished GSH synthesis in OA chondrocytes impairs cellular antioxidant capacity, sensitizing cells to copper toxicity; notably, TTM attenuates cuproptosis by enhancing GSH expression and preserving mitochondrial integrity (Wang et al., 2023e).

Regarding ECM metabolism, cuproptosis suppresses anabolic genes (COL2A1, ACAN) while upregulating catabolic enzymes (MMP-3, MMP-13, ADAMTS-5), disrupting the synthesis-degradation equilibrium and accelerating cartilage structural deterioration (Che et al., 2024). This process is aggravated by cuproptosis-triggered CRG dysregulation linked to OA cartilage ECM degradation and is more evident in advanced KOA (Chang et al., 2023). In the synovial compartment, cuproptosis in fibroblast-like synoviocytes promotes cell death and releases DAMPs and inflammatory mediators, fueling a self-perpetuating “inflammation-cuproptosis-cartilage destruction” cycle (Deng et al., 2024). Immune infiltration analyses reveal significant associations between CRG expression profiles and macrophage/T cell recruitment in synovial tissue, underscoring cuproptosis as a bridge between metal dyshomeostasis and inflammatory cascades (Shen et al., 2025).

#### 3.1.3 Therapeutic strategies targeting cuproptosis in KOA

Copper chelators, such as TTM and D-penicillamine, effectively sequester intracellular excess copper, thereby attenuating copper overload, suppressing FDX1-mediated cuproptosis, and preserving chondrocyte viability. Specifically, TTM administration significantly reduces copper levels in KOA-derived chondrocytes and joint tissues, downregulates FDX1 expression, inhibits lipoylated TCA cycle protein aggregation (e.g., DLAT), and concurrently upregulates cartilage anabolic markers COL2A1 and ACAN while suppressing matrix-degrading enzymes (e.g., MMP13, ADAMTS5), thereby mitigating cartilage degeneration and synovial inflammation (Hua et al., 2025).

Genetic modulation of cuproptosis regulators offers another strategic avenue. Silencing pro-cuproptotic factors (e.g., FDX1, LIAS, DLAT) via siRNA or CRISPR-based approaches significantly reduces copper-induced chondrocyte death and extracellular matrix degradation (Wang et al., 2023a; Chen X. et al., 2024). Conversely, enhancing expression of copper efflux transporters (e.g., ATP7A) or endogenous antioxidants bolsters cellular resistance to copper toxicity.

Antioxidant therapies—including N-acetylcysteine (NAC), curcumin, and resveratrol—counteract cuproptosis by scavenging copper-induced ROS, preserving mitochondrial function, and inhibiting lipoylated protein oligomerization. Curcumin, for instance, modulates FDX1 and GPX4 expression and synergistically enhances copper chelation efficacy (Cao et al., 2025).

However, current copper chelators (TTM, D-penicillamine) lack joint specificity and may cause systemic copper deficiency with long-term use. Their intra-articular half-life is short, necessitating frequent injections. Nanoparticle-based copper chelator delivery systems are under preclinical investigation but have not yet entered clinical trials for KOA.

### 3.2 Ferroptosis in KOA

#### 3.2.1 Dysregulation of ferroptosis in KOA

Aberrant activation of ferroptosis plays a pivotal role in KOA pathogenesis, characterized by disrupted iron homeostasis, dysregulated expression of ferroptosis-related molecular regulators, and a strong correlation with the severity of articular cartilage degeneration. In early KOA, mild TFR1 upregulation and moderate GPX4 reduction are observed, with partial compensation by the FSP1-CoQ10 axis. In advanced KOA, marked iron overload, SLC7A11/GPX4 suppression, and ACSL4 upregulation are typical. Clinical evidence reveals significantly elevated iron ion concentrations in the synovial fluid and cartilage tissues of KOA patients compared to healthy controls, with levels positively correlating with radiographic severity assessed by the Kellgren-Lawrence (KL) grading system (Zhou et al., 2026).

At the molecular level, KOA chondrocytes exhibit a distinct ferroptosis-related signature: upregulation of TFR1/TFRC enhances cellular iron uptake, while downregulation of FPN1 and FTH1 impairs iron export and storage, collectively promoting labile iron pool accumulation (He et al., 2023). Concurrently, suppression of SLC7A11 and GPX4 compromises the antioxidant defense system, leading to uncontrolled accumulation of lipid peroxides such as malondialdehyde (MDA) and 4-hydroxynonenal (4-HNE) (Lv Z. et al., 2022). Additionally, elevated expression of ACSL4 and lipoxygenases (LOXs, including ALOX15) accelerates the esterification of PUFAs into membrane phospholipids and their subsequent peroxidation, thereby amplifying ferroptotic vulnerability (Zhang et al., 2024).

Cell-type specifically: in chondrocytes, ferroptosis is the dominant death pathway under mechanical overload. In synovial macrophages, ferroptosis promotes IL-1β and TNF-α release. In subchondral osteoblasts, ferroptosis may impair bone formation, contributing to abnormal subchondral bone remodeling.

#### 3.2.2 Mechanisms of ferroptosis regulation in KOA

Central to this process is the disruption of iron homeostasis, which triggers chondrocyte ferroptosis and concurrently exacerbates ECM degradation and synovial inflammation (Xu et al., 2023; Lu et al., 2024). In chondrocytes, excessive labile iron accumulation catalyzes Fenton reactions, generating hydroxyl radicals (·OH) that peroxidize PUFAs within plasma membranes, leading to cell death (Zhang L. et al., 2026). Critically, lipid peroxidation products further amplify ROS production, establishing a self-amplifying “ROS–lipid peroxidation–ferroptosis” cycle that intensifies chondrocyte damage (Cao et al., 2023b). Concurrently, dysfunction of system Xc^−^ (notably SLC7A11) and downregulation of GPX4 impair cellular antioxidant capacity, accelerating ferroptotic progression. Experimental evidence confirms that ferroptosis inhibitors (e.g., Ferrostatin-1, liproxstatin-1) or GPX4 activators significantly attenuate IL-1β- or ferric ammonium citrate (FAC)-induced chondrocyte injury and cartilage degradation *in vitro* and in DMM mouse models (Cao et al., 2024).

Regarding ECM metabolism, ferroptosis-derived ROS activate pro-inflammatory and catabolic signaling cascades. Elevated ROS stimulate NF-κB pathways, suppressing anabolic genes (e.g., COL2A1, ACAN) while upregulating matrix-degrading enzymes such as MMP13 and ADAMTS-5, thereby accelerating cartilage destruction (Gong et al., 2023).

In synovial pathology, ferroptosis in synovial macrophages and fibroblasts promotes the release of TNF-α, IL-6, and other inflammatory mediators, fueling a vicious “inflammation–ferroptosis–cartilage damage” cycle that sustains low-grade synovitis and joint degeneration (Yan et al., 2024; Xia and Gong, 2022; Zhang W. et al., 2026). Additionally, iron deposition in subchondral bone and synovium highlights the systemic nature of iron dysregulation across the “cartilage–bone–synovium” axis (Zhou et al., 2026).

#### 3.2.3 Therapeutic strategies targeting ferroptosis in KOA

Iron chelators—including deferoxamine (DFO), deferiprone, and lactoferrin—sequester intracellular labile iron pools, attenuate iron-catalyzed lipid peroxidation, and preserve chondrocyte viability. DFO significantly reduces intracellular iron accumulation and MDA levels in IL-1β- or FAC-stimulated chondrocytes, suppresses ferroptotic death, enhances ECM synthesis (e.g., type II collagen), and inhibits catabolic enzymes such as MMP13, thereby mitigating cartilage degradation in DMM mouse models.

Antioxidant agents and specific ferroptosis inhibitors—including Ferrostatin-1 (Fer-1), liproxstatin-1 (Lip-1), vitamin E, and NAC—scavenge ROS, block phospholipid peroxidation, and restore redox homeostasis. Fer-1 markedly decreases lipid peroxidation products, upregulates key ferroptosis defense proteins (SLC7A11 and GPX4), reduces chondrocyte death, and alleviates synovitis in preclinical OA models (Cao et al., 2024). Genetic modulation further validates this axis: cartilage-specific GPX4 knockout exacerbates experimental OA, whereas overexpression of GPX4 or SLC7A11, or silencing of pro-ferroptotic genes (e.g., TFR1, ACSL4), robustly suppresses chondrocyte ferroptosis and attenuates joint pathology (Wang S. et al., 2022; Pang et al., 2024).

Natural compounds offer multi-targeted, low-toxicity therapeutic potential. Curcumin ameliorates IL-1β-induced chondrocyte ferroptosis by enhancing GPX4 expression and reducing ROS/MDA levels. Resveratrol modulates ferroptosis via upregulation of GPX4, SLC7A11, and TFRC, while concurrently regulating immune cell infiltration in synovium (Song C. et al., 2025). Additional phytochemicals such as icariin activate the SLC7A11/GPX4 signaling axis to inhibit ferroptosis (Xiao et al., 2024), and astragaloside IV (AS-IV) downregulates p53 and MMP13 while elevating SOX9, collagen II, and GSH, demonstrating chondroprotective efficacy in OA rat models. Emerging nanotherapeutic platforms, including ROS-responsive nanoparticles and carbon dots, enhance targeted delivery of ferroptosis inhibitors to chondrocytes, improving ECM preservation and reducing off-target effects (Wang D. et al., 2025).

Despite these promising results, most ferroptosis-targeting agents have not advanced beyond preclinical stages. Key limitations include poor bioavailability, lack of joint specificity, and unknown long-term safety. Clinical trials are urgently needed.

### 3.3 Apoptosis in KOA

#### 3.3.1 Dysregulation of apoptosis in KOA

Extensive clinical evidence confirms that the apoptotic rate of chondrocytes in KOA-affected cartilage is significantly elevated compared to healthy controls, exhibiting a positive correlation with the severity of histological cartilage damage. In advanced-stage KOA, increased numbers of apoptotic chondrocytes, hypocellularity, and thinning or focal absence of the chondrocyte layer are consistently observed (Charlier et al., 2016). TUNEL assays further validate higher apoptosis levels in degraded cartilage regions, correlating with reduced Safranin O staining intensity and elevated OARSI scores (Gu et al., 2024).

Stage-dependent pattern: In early KOA, apoptosis is mainly confined to the superficial zone and remains at low basal levels. In middle and advanced KOA, extensive apoptosis is seen in middle and deep zones, with high caspase-3 activation. Molecular analyses reveal dysregulation of key apoptotic regulators in KOA chondrocytes. In the extrinsic pathway, upregulated expression of death receptors (Fas, TNFR1) and their ligands (FasL, TNF-α), alongside increased caspase-8 activation, has been documented. Concurrently, the intrinsic pathway is prominently activated: pro-apoptotic proteins (Bax, Bak, Bad) are elevated, while anti-apoptotic members (Bcl-2, Bcl-XL) are suppressed, leading to mitochondrial membrane depolarization, cytochrome c release, and sequential activation of caspase-9 and executioner caspase-3 (Miao et al., 2019; Zhu W. et al., 2024; Liu et al., 2019). Notably, p53 is significantly upregulated in OA chondrocytes and functions as a critical transcriptional activator of the intrinsic pathway (Kobayashi et al., 2016). Additional contributors include ROS and nitric oxide (NO), which induce oxidative stress and trigger both caspase-dependent and caspase-independent apoptosis (Xiao et al., 2023).

Cell-type specificity: Apoptosis occurs not only in chondrocytes but also in synovial fibroblasts (promoting inflammation) and subchondral osteoblasts (contributing to bone loss). In synovial macrophages, apoptosis may be either protective (clearing activated cells) or detrimental (releasing DAMPs), depending on context.

#### 3.3.2 Mechanisms of apoptosis regulation in KOA

In chondrocyte injury, multiple pathological stimuli converge on apoptotic signaling. Mechanical overload directly triggers the intrinsic pathway via mechanosensitive ion channels (e.g., TRPV4 and Piezo1), inducing mitochondrial dysfunction, cytochrome c release, and sequential activation of caspase-9 and caspase-3. Pro-inflammatory cytokines—including IL-1β and TNF-α—activate the extrinsic pathway through death receptor engagement and caspase-8 initiation, while concurrently stimulating ROS and NO production to amplify intrinsic apoptosis (Xiao et al., 2023). ROS accumulation further damages mitochondria, upregulates pro-apoptotic Bax, suppresses anti-apoptotic Bcl-2, and enhances death receptor expression, creating bidirectional crosstalk between apoptotic pathways (Li et al., 2019; Zhuang et al., 2020).

Regarding ECM metabolism, apoptotic chondrocytes exhibit severely impaired synthesis of type II collagen and aggrecan. Concurrently, apoptosis-associated proteases (e.g., MMP-1, MMP-13) and caspase-mediated cleavage events accelerate ECM degradation (Fa et al., 2021). Critically, dying chondrocytes release DAMPs, inflammatory cytokines, and apoptotic bodies that activate synovial macrophages and fibroblasts, perpetuating synovitis (Deng et al., 2024). In turn, synovium-derived cytokines reinforce chondrocyte apoptosis via NF-κB and MAPK signaling cascades. This “inflammation-apoptosis-ECM destruction” vicious cycle is further potentiated by impaired efferocytosis in OA synovium, leading to secondary necrosis and amplified inflammation.

#### 3.3.3 Therapeutic strategies targeting apoptosis in KOA

Caspases, particularly caspase-3, serve as central executioners in chondrocyte apoptosis; their inhibition attenuates cartilage matrix degradation and supports ECM homeostasis (Zhu W. et al., 2024; Xiao et al., 2023). Pharmacological or genetic suppression of caspase activity has been shown to reduce chondrocyte apoptosis and promote expression of cartilage-specific markers such as COL2A1 and aggrecan in experimental models (Chen et al., 2022).

Modulation of the Bcl-2 protein family constitutes another pivotal strategy. Overexpression of anti-apoptotic proteins (e.g., Bcl-2, Bcl-xL) or silencing of pro-apoptotic members (e.g., Bax, Bad) shifts the mitochondrial balance toward cell survival. Elevated Bax expression correlates with increased caspase-3 activation and chondrocyte apoptosis in OA progression, whereas Bcl-2 upregulation exerts protective effects (Zhang H. et al., 2025).

Complementary strategies further enhance therapeutic efficacy:

1. Anti-inflammatory interventions: Agents targeting TNF-α or NF-κB signaling reduce inflammatory mediators, thereby attenuating apoptosis initiation (Luo et al., 2021).
2. Antioxidant approaches: Scavenging ROS mitigates oxidative stress-induced intrinsic apoptosis, preserving mitochondrial integrity (Xiao et al., 2023).
3. Growth factor therapy: TGF-β and IGF-1 promote chondrocyte survival by upregulating anti-apoptotic signals and stimulating ECM synthesis (Yan and Wu, 2020).
4. Natural compounds and traditional medicine: Plant-derived extracts and microRNA-based strategies demonstrate dual regulation of apoptosis and inflammation (Wang Z. et al., 2022).

### 3.4 Autophagy in KOA

#### 3.4.1 Dysregulation of autophagy in KOA

Autophagy exhibits a context-dependent dual role in KOA, with its activity dynamically modulated across disease stages and microenvironmental cues. In early KOA, autophagy is upregulated as a cytoprotective response (elevated LC3-II, Beclin-1; suppressed mTOR) to maintain chondrocyte homeostasis, mitigate oxidative stress, and suppress apoptosis. In moderate-to-advanced KOA, autophagy becomes dysregulated: activity is often diminished due to age-related decline or pathological inhibition, leading to accumulation of damaged organelles and proteins; in specific contexts, excessive or maladaptive autophagy may trigger autophagic cell death, further accelerating cartilage degradation. Clinical studies report elevated expression of autophagy markers in articular cartilage of early-stage KOA patients, correlating with preserved chondrocyte viability (Cheng et al., 2017; Cheng et al., 2016). Conversely, histopathological analyses reveal reduced Beclin-1 and LC3-II levels alongside increased chondrocyte apoptosis and matrix degradation in late-stage human KOA cartilage (Zhao et al., 2025; He et al., 2026).

Cell-type specificity: In synovial fibroblasts, autophagy is often downregulated in fibrotic synoviocytes, contributing to synovial fibrosis. In synovial macrophages, impaired autophagic clearance leads to inflammasome activation. In subchondral bone cells, the role of autophagy is controversial, with some studies suggesting protective effects and others indicating autophagic cell death.

#### 3.4.2 Mechanisms of autophagy regulation in KOA

In early-stage KOA, basal autophagy functions protectively by selectively removing damaged mitochondria (via mitophagy), misfolded proteins, and ROS, thereby preserving chondrocyte viability and metabolic function (Mei et al., 2026; Tang et al., 2024). This process mitigates oxidative stress and sustains cellular energy homeostasis, which is critical for chondrocytes residing in an avascular, hypoxic microenvironment (Arias et al., 2020; D'Adamo et al., 2017). Concurrently, functional autophagy suppresses catabolic enzyme expression (e.g., MMP-1, MMP-13, ADAMTS-5) and enhances anabolic activity (e.g., type II collagen synthesis), contributing to ECM stability (Huang et al., 2019; Wang A. et al., 2022; Li Y. et al., 2024).

Conversely, in moderate-to-advanced KOA, autophagic dysfunction emerges as a key pathological driver. Age-related or stress-induced decline in autophagic activity leads to accumulation of dysfunctional organelles, protein aggregates, and excessive ROS, triggering mitochondrial failure, chondrocyte senescence, apoptosis, and accelerated cartilage destruction (Jeon and Im, 2017). Notably, impaired mitophagy specifically exacerbates chondrocyte degeneration due to unresolved mitochondrial damage. Paradoxically, excessive or dysregulated autophagy under severe stress may induce autophagic cell death, degrading essential cellular components and further compromising chondrocyte function.

#### 3.4.3 Therapeutic strategies targeting autophagy in KOA

Targeted modulation of autophagy represents a promising therapeutic strategy for KOA, with the core principle being the restoration and maintenance of balanced autophagic activity tailored to disease stage. Therefore, stage-specific intervention is critical—moderate activation of autophagy is advocated in early KOA to counteract age- or stress-induced autophagy decline, while in subsets of mid-to-late KOA characterized by pathological autophagy overactivation, transient inhibition may be warranted to prevent autophagic cell death (Goutas et al., 2018).

Pharmacological autophagy inducers, such as rapamycin (an mTOR inhibitor) and metformin (an AMPK activator), enhance cytoprotective autophagy primarily by suppressing the PI3K/AKT/mTOR pathway and/or activating the AMPK/mTOR axis. Rapamycin promotes clearance of ROS and damaged mitochondria via mitophagy, reduces chondrocyte apoptosis, stimulates extracellular matrix synthesis, and attenuates cartilage degeneration in preclinical KOA models (Wan et al., 2019). Similarly, metformin and other AMPK activators (e.g., geniposide, polydatin) exert chondroprotection by stimulating autophagy flux through AMPK-dependent mTOR inhibition (Huang et al., 2023).

Conversely, in contexts where excessive autophagy contributes to chondrocyte loss—observed in certain advanced KOA phenotypes—transient application of autophagy inhibitors such as 3-methyladenine (3-MA) or chloroquine may be beneficial. 3-MA, by blocking autophagosome formation via class III PI3K inhibition, reduces autophagy-dependent cell death and mitigates cartilage damage in experimental models (Cheng et al., 2016; Tao et al., 2020).

Natural compounds offer multi-targeted autophagy regulation with favorable safety profiles. Curcumin activates autophagy via PI3K/AKT/mTOR inhibition, reducing synovial fibrosis and cartilage damage (He et al., 2026). Resveratrol enhances chondrocyte autophagy and viability under oxidative stress, while saikosaponin D suppresses inflammation and restores autophagy by inhibiting PI3K/AKT/mTOR signaling (Qin et al., 2017). Quercetin and other phytochemicals similarly modulate AMPK/mTOR pathways to promote cartilage integrity.

### 3.5 Stage-dependent and cell-type-specific characteristics of the four pathways in KOA

The four pathways exhibit dynamic changes across KOA progression and differ among cell types within the joint. (Table 2).

**TABLE 2:** Stage-dependent and cell-type-specific features of cuproptosis, ferroptosis, apoptosis, and autophagy in KOA.

Pathway	Cuproptosis	Ferroptosis	Apoptosis	Autophagy
Early KOA	Mild ↑ FDX1, preserved GSH	Mild ↑ TFR1, ↓ GPX4	Low basal, mainly in superficial zone	↑ protective (LC3-II↑, mTOR↓)
Middle KOA	Moderate ↑ copper, ↓ ATP7A	↑ Iron, ↓ SLC7A11	Moderate ↑ Bax/Bak	Variable (often ↓ Beclin-1)
Advanced KOA	Marked ↑ FDX1/DLAT, GSH depletion	Severe lipid peroxidation, ACSL4↑	High caspase-3 activation, extensive cell loss	↓ or maladaptive ↑ (autophagic cell death)
Chondrocytes	↑ Sensitivity, especially in superficial zone	High sensitivity under mechanical overload	↑ in superficial and middle zones	↓ with age and disease severity
Synovial fibroblasts	↑ CTR1, cuproptosis → IL-6/TNF-α	Moderate sensitivity	↑ in inflamed synovium	↓ in fibrotic synoviocytes
Synovial macrophages	ND	↑ Ferroptosis → IL-1β/TNF-α	↑ Macrophage apoptosis	↓ Impaired clearance
Subchondral bone cells	? (limited data)	↑ Ferroptosis → impaired bone formation	↑ Osteoblast apoptosis	↑? Autophagic cell death (controversial)
ND: not determined; ?: conflicting or insufficient evidence				

## 4 Integrated crosstalk network and causal regulatory mechanisms in KOA

Cuproptosis, ferroptosis, apoptosis, and autophagy do not operate in isolation during KOA pathogenesis; rather, they engage in intricate crosstalk mediated by shared regulatory nodes—including ROS generation, GSH metabolism, and intersecting signaling pathways—collectively governing chondrocyte fate and articular cartilage degeneration.

### 4.1 GSH and ROS as central hubs with causal hierarchy

Central to this network is GSH homeostasis. GSH functions as a critical redox buffer: it chelates excess copper to suppress cuproptosis (Shi et al., 2024), serves as an essential cofactor for GPX4 to inhibit lipid peroxidation-driven ferroptosis, scavenges ROS to attenuate apoptosis initiation, and sustains balanced autophagic flux. In KOA chondrocytes, diminished GSH synthesis (e.g., via downregulation of SLC7A11) or heightened consumption disrupts this equilibrium, sensitizing cells to cuproptosis and ferroptosis while permitting ROS accumulation that triggers apoptotic pathways and dysregulates autophagy (either suppression or maladaptive hyperactivation). Conversely, activation of cuproptosis or ferroptosis further depletes GSH and amplifies ROS, establishing a self-reinforcing vicious cycle that accelerates chondrocyte injury (Wang et al., 2023f).

Importantly, GSH depletion is an early causal event that precedes overt cell death. This hierarchical relationship (metal excess → GSH depletion → ROS ↑ → pathway activation) has been validated in recent studies using GSH synthesis inhibitors and supplementation experiments.

ROS acts as a pivotal signaling hub in this crosstalk. Copper overload (via FDX1-mediated lipoylation) and iron-driven Fenton reactions generate mitochondrial ROS, while defective autophagy or apoptotic stress further elevates oxidative burden (Malla et al., 2025). Elevated ROS reciprocally promotes cuproptosis (via FDX1 upregulation), ferroptosis (by suppressing GPX4 activity and upregulating transferrin receptor TFR1), intrinsic apoptosis (through mitochondrial membrane permeabilization and cytochrome c release), and extrinsic apoptosis (via death receptor sensitization). ROS also modulates autophagy in a dose-dependent manner: moderate levels activate AMPK and inhibit mTOR to induce protective autophagy, whereas excessive ROS impairs lysosomal function and autophagic clearance, exacerbating cellular damage (Tang et al., 2024).

### 4.2 Key signaling pathways integrating the four processes

**FIGURE 5:**
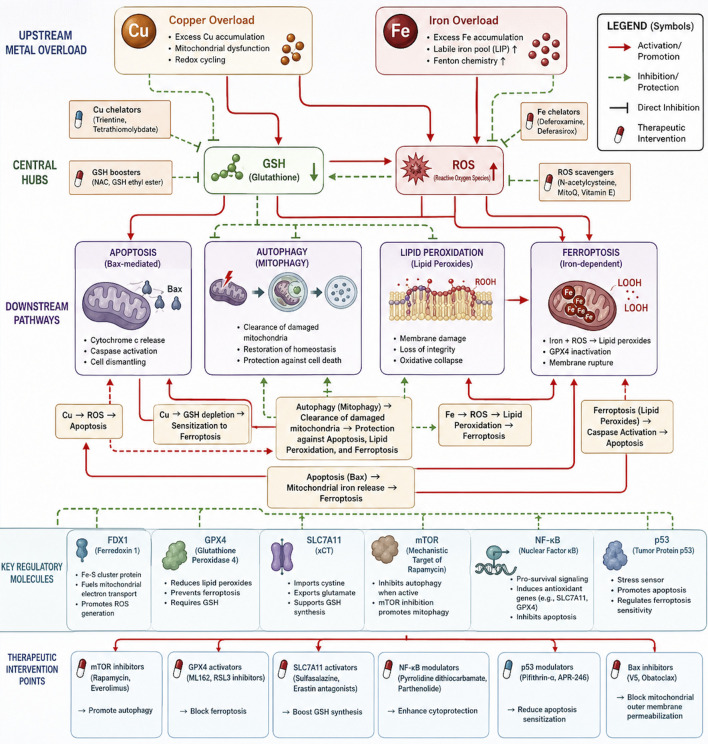
Schematic representation of the integrated crosstalk network between cuproptosis, ferroptosis, apoptosis, and autophagy in KOA. Central hubs: GSH (green) and ROS (red). Upstream: Copper (Cu) and Iron (Fe) overload. Downstream: Four pathways. Crosstalk arrows: Cu → GSH depletion → sensitization to ferroptosis; Cu → ROS → apoptosis; Fe → ROS → lipid peroxidation → ferroptosis; Autophagy (mitophagy) → clearance of damaged mitochondria → protection against all three death pathways; Apoptosis (Bax) → mitochondrial iron release → ferroptosis; Ferroptosis (lipid peroxides) → caspase activation → apoptosis. Key regulatory molecules: FDX1, GPX4, SLC7A11, mTOR, NF-κB, p53. Therapeutic intervention points indicated with pharmaceutical symbols.

The mTOR pathway critically regulates autophagy and intersects with metal-dependent death pathways: mTOR activation suppresses autophagy while promoting expression of cuproptosis (FDX1), ferroptosis (via GPX4 inhibition), and apoptosis-related proteins (caspases); mTOR inhibition exerts the opposite effects. Similarly, NF-κB—activated by ROS and inflammatory cytokines (e.g., IL-1β)—upregulates pro-apoptotic factors, cuproptosis/ferroptosis drivers (e.g., ATP7B, GLS), and inflammatory mediators while suppressing cytoprotective autophagy, thereby amplifying cartilage destruction (Shen et al., 2025) (Figure 5).

p53, upregulated in OA chondrocytes, promotes apoptosis (via Bax and Puma) and ferroptosis (via SLC7A11 repression) (Ma et al., 2024). Emerging evidence suggests p53 may also influence cuproptosis sensitivity through metabolic regulation (Xiong et al., 2023).

### 4.3 Functional interdependencies among the four pathways

1. Autophagy → mitophagy clears damaged mitochondria, limiting ROS and protecting against cuproptosis, ferroptosis, and apoptosis (Xie Y. et al., 2025; Wang H. et al., 2025); however, autophagic dysfunction (common in aged or OA chondrocytes) accelerates all three death pathways (Zhao et al., 2025).
2. Apoptosis ↔ ferroptosis exhibit bidirectional potentiation: Bax-mediated mitochondrial iron release promotes ferroptosis, while ferroptotic lipid peroxides (e.g., MDA) activate caspase-dependent apoptosis.
3. Cuproptosis → ferroptosis synergize via GSH depletion and ROS amplification; copper-induced GSH synthesis disorder (e.g., via GLS dysregulation) sensitizes cells to ferroptosis (Wang et al., 2023f).
4. Cuproptosis → apoptosis: Copper-induced ROS directly activate apoptotic cascades, linking cuproptosis to apoptosis (Xu et al., 2022).

### 4.4 A unified network model

The causal hierarchy begins with metal (copper/iron) overload, leading to GSH depletion and ROS accumulation, which then simultaneously activate cuproptosis (FDX1-dependent), ferroptosis (GPX4 inhibition), apoptosis (caspase activation), and autophagy dysregulation (either insufficient or excessive). Therapeutic intervention points include copper chelators, iron chelators, GPX4 activators, caspase inhibitors, and autophagy modulators (rapamycin, 3-MA) (Figure 5).

**FIGURE 6:**
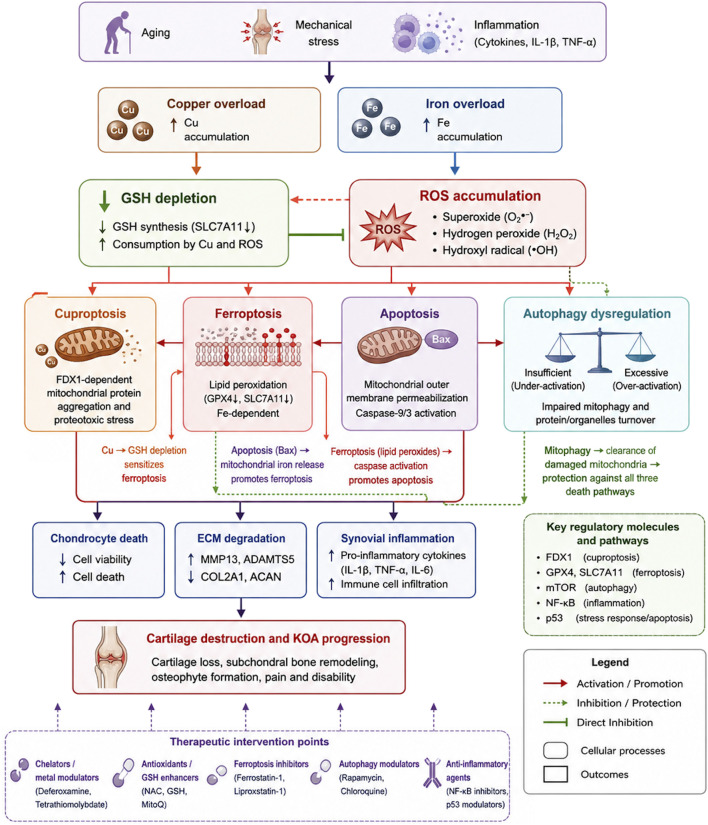
Schematic representation of the integrated pathological cascade from dysregulation of metal homeostasis to cartilage destruction in KOA. Flow diagram starting from top: Aging, mechanical stress, and inflammation lead to copper and iron overload in the joint. This causes GSH depletion and ROS accumulation, which simultaneously activate cuproptosis (mitochondrial protein aggregation), ferroptosis (lipid peroxidation), apoptosis (caspase activation), and autophagy dysregulation (either insufficient protective autophagy or excessive autophagic cell death). These four processes converge to promote chondrocyte death, ECM degradation (↑MMP13, ↓COL2A1), and synovial inflammation, ultimately resulting in cartilage destruction and KOA progression.

The entire pathological cascade, from dysregulation of metal homeostasis to GSH/ROS imbalance, activates four pathways simultaneously and ultimately leads to cartilage destruction (Figure 6).

## 5 Current issues, translational bottlenecks, and future directions

Summary of key molecular alterations and therapeutic strategies targeting four cell death pathways in KOA (Table 3).

**TABLE 3:** Key molecular alterations and therapeutic strategies targeting the 4 cell death pathways in KOA.

Cell death pathway	Key molecular alterations	Therapeutic strategy	Representative agents/Methods	Evidence level	Major limitations
Cuproptosis	FDX1↑, DLAT↑, ATP7A↓	Copper chelation, antioxidant therapy	TTM, quercetin, NAC	Preclinical (*in vitro*/rodent)	Lack joint specificity, systemic copper deficiency risk
Ferroptosis	TFR1↑, SLC7A11↓, GPX4↓	Iron chelation, inhibition of lipid peroxidation	DFO, Ferrostatin-1, Liproxstatin-1	Preclinical (*in vitro*/rodent)	Poor bioavailability, short half-life
Apoptosis	Bax↑, Bcl-2↓, Caspase-3↑	Caspase inhibition, Bcl-2 upregulation	z-VAD-fmk, curcumin	Preclinical (*in vitro*)	Limited translation to human OA
Autophagy	LC3-II↑/↓, p62↑/↓	Stage-specific modulation	Rapamycin (early stage), 3-MA (late stage)	Preclinical (rodent models)	Threshold of protective vs. pathological unclear

### 5.1 Current research controversies and unresolved gaps

Current research on cuproptosis, ferroptosis, apoptosis, and autophagy in KOA has advanced our understanding of chondrocyte pathophysiology; however, significant translational gaps remain. Specifically:

The protective vs. pathological threshold of autophagic flux in KOA remains undefined. What level of LC3-II conversion separates cytoprotection from autophagic cell death? This is a major controversy in the field. Autophagy exhibits a context-dependent dual role in OA—cytoprotective under basal stress but pathogenic upon dysregulation (Huang et al., 2025), and literature explicitly distinguishes “autophagy-mediated cell death” from “autophagy-dependent cell death” mechanisms, yet quantitative thresholds for LC3-II conversion or flux transition remain unestablished.

Most crosstalk evidence is correlative; causal validation (e.g., using conditional knockouts of FDX1, GPX4, or ATG7 in specific cell types) is lacking. For example, it is unclear whether cuproptosis directly causes ferroptosis or merely co-occurs. Bioinformatics analyses reveal significant co-expression between ferroptosis-related genes (e.g., ATF3, ACSL1) and autophagy-related genes (e.g., IL6, NFKBIA) in TNF-α-induced chondrocyte inflammation models (Wang C. et al., 2026), yet causal mechanisms remain undefined; reviews note that “the potential interplay mechanisms between autophagy and ferroptosis remain unelucidated” (Wang J. G.et al., 2026) and “crosstalk between cuproptosis and ferroptosis is obscure” (Wang et al., 2023f).

Cuproptosis studies in KOA are limited to bioinformatics and a few preclinical experiments; no large-scale human validation exists for CRG biomarkers. The causal sequence of metal dysregulation (e.g., does copper overload precede iron overload?) has not been established in longitudinal human cohorts. Recent reviews identify cuproptosis as a potential OA driver linked to copper dyshomeostasis and propose therapeutic strategies (e.g., Cu chelation, antioxidant reinforcement) (Shen et al., 2025), yet validation relies on GEO dataset integration and *in vitro* models (e.g., quercetin suppressing cuproptosis via ATP7B/GLS modulation) (Shi et al., 2024); no longitudinal human cohort data clarify temporal sequences of copper/iron dysregulation.

The interplay between these pathways and other disease modifiers (mechanical loading, gut microbiome, systemic metabolic health) remains underexplored. Although OA pathogenesis involves multifactorial etiology where chondrocyte death pathways (apoptosis, pyroptosis, autophagy, ferroptosis, cuproptosis) interact with oxidative stress and inflammation, current studies rarely integrate metal-dependent cell death mechanisms with systemic modifiers. Emerging evidence links gut microbiome metabolites and metabolic disorders to ferroptosis/cuproptosis in other contexts, but OA-specific investigations into mechanical loading, microbiome, or metabolic health interactions are scarce (Huang et al., 2025).

### 5.2 Translational bottlenecks and safety concerns

1. Copper/iron chelators (TTM, DFO) lack joint specificity and risk systemic metal deficiency (e.g., TTM can cause copper deficiency anemia; DFO may cause iron deficiency).
2. Nanoparticle systems (ROS-responsive, MOF-based) show promise but face challenges in large-scale manufacturing, long-term safety, and regulatory approval. No nano-formulation has yet entered clinical trials for KOA.
3. Poor bioavailability and short intra-articular half-life of natural compounds (curcumin, resveratrol) remain unresolved despite extensive preclinical efficacy. Direct intra-articular injection may overcome this but is invasive and requires repeated administration.
4. Combination therapies (e.g., chelator + autophagy inducer) have not been tested *in vivo* for KOA, and potential drug-drug interactions are unknown.
5. Lack of validated biomarkers for patient stratification: synovial fluid levels of SLC7A11, GPX4, FDX1, LC3-II, and copper/iron have been proposed, but none have been validated in multi-center longitudinal studies.

### 5.3 Future research directions

Given the current state of research, future studies could focus on the following areas.

1. Single-cell multi-omics (transcriptomics, proteomics, metabolomics) to map pathway activity in individual chondrocytes, synoviocytes, and bone cells across early, middle, and advanced KOA stages. This will identify core regulatory factors and crosstalk targets with cell-type resolution.
2. Conditional knockout animal models (e.g., chondrocyte-specific GPX4 knockout, FDX1 knockout, ATG7 knockout) to establish causal roles of each pathway in KOA pathogenesis and to determine the hierarchy of crosstalk.
3. Stage-specific therapeutic algorithms based on Table 4 profiles. For example,:

**TABLE 4:** Systematic classification of therapeutic agents targeting cuproptosis, ferroptosis, apoptosis, and autophagy in KOA.

Agent class	Specific targets	Mechanism	Evidence level	Advantages	Limitations	Safety risks
Copper chelators (TTM, D-penicillamine)	Cu^2+^/Cu^+^	Sequester excess copper, inhibit FDX1/DLAT aggregation	*In vitro*, rodent KOA models	Directly blocks cuproptosis	Lack joint specificity, short intra-articular half-life	Systemic copper deficiency, anemia
Iron chelators (DFO, deferiprone)	Fe^2+^/Fe^3+^	Reduce labile iron pool, inhibit Fenton reaction	*In vitro*, rodent KOA models	Attenuates ferroptosis	Poor oral bioavailability, repeated injections required	Iron deficiency, local irritation
Ferroptosis inhibitors (Fer-1, Lip-1)	Lipid peroxides, GPX4	Scavenge ROS, restore GPX4 activity	*In vitro*, rodent KOA models	Highly specific	Not clinically approved for KOA	Unknown long-term safety
Autophagy inducers (rapamycin, metformin)	mTOR, AMPK	Activate protective autophagy	Rodent KOA models	Stage-specific benefit in early KOA	May exacerbate late-stage disease	Immunosuppression (rapamycin)
Natural compounds (curcumin, resveratrol, quercetin)	Multiple (GSH, ROS, FDX1, GPX4)	Pleiotropic: antioxidant, metal chelation, autophagy modulation	*In vitro*, rodent models	Multi-target, low toxicity	Poor bioavailability, rapid metabolism	Generally safe but variable efficacy
Nanoparticle systems (ROS-responsive, MOF)	Joint-specific delivery	Enhance targeting, sustained release	Preclinical (*in vivo*)	Improved joint accumulation	Manufacturing challenges, regulatory hurdles	Unknown long-term biocompatibility

Early KOA with low autophagic flux → autophagy inducers (rapamycin, metformin).

Advanced KOA with high caspase-3 → apoptosis inhibitors (z-VAD-fmk).

Ferroptosis-dominant phenotype (low GPX4, high TFR1) → iron chelators + Fer-1.

Cuproptosis-dominant phenotype (high FDX1, low ATP7A) → copper chelators + NAC.

4. Clinical trials of combination therapies (e.g., TTM + Fer-1 + rapamycin) with careful safety monitoring, using intra-articular injection and nanocarrier delivery to enhance joint specificity.
5. Biomarker development: large-scale, longitudinal clinical cohorts to validate synovial fluid or serum levels of SLC7A11, GPX4, FDX1, LC3-II, and copper/iron as diagnostic, prognostic, and predictive biomarkers.
6. Natural product screening and mechanism elucidation: identify novel compounds that simultaneously modulate multiple pathways (e.g., GSH restoration, ROS scavenging, autophagy regulation) with favorable safety profiles.

Summary of systematic classifications of therapeutic agents, including their specific targets, mechanisms, levels of evidence, and safety risks (Table 4).

## 6 Discussion

### 6.1 Clinical implications of targeting cell death pathways in KOA

The four forms of programmed cell death and autophagy discussed in this review—cuproptosis, ferroptosis, apoptosis, and autophagy—represent interconnected pathological processes that collectively drive cartilage degeneration in knee osteoarthritis. While each pathway possesses distinct molecular signatures and morphological features, their functional integration through shared metabolic and signaling networks underscores the complexity of chondrocyte fate regulation in the osteoarthritic joint.

From a clinical perspective, targeting these pathways offers several opportunities.

1. Biomarker potential: Synovial fluid or serum levels of SLC7A11, GPX4, FDX1, LC3-II, and copper/iron could serve as diagnostic or prognostic markers. For example, low GPX4 and high TFR1 may indicate a ferroptosis-dominant disease phenotype, guiding iron chelation therapy. High FDX1 and low ATP7A may indicate cuproptosis sensitivity, guiding copper chelation.
2. Patient stratification: Stage-specific pathway activity profiles (Table 4) could guide personalized therapy. Early KOA with low autophagic flux might benefit from rapamycin, while advanced KOA with high caspase-3 might require apoptosis inhibitors.
3. Multi-target DMOADs: Simultaneous targeting of cuproptosis and ferroptosis via GSH restoration (e.g., NAC + curcumin), or combined autophagy enhancement with apoptosis blockade, represents a rational strategy that has not yet been clinically tested. The convergence of these pathways on GSH and ROS suggests that interventions restoring redox balance may have broad-spectrum chondroprotective effects.
4. Unmet need: Current clinical management of KOA lacks disease-modifying drugs; symptom relief with NSAIDs or analgesics does not halt structural progression. Targeting integrated cell death pathways offers a new avenue for DMOAD development.

### 6.2 Other key insights

A key insight emerging from this synthesis is that GSH depletion and ROS accumulation serve as common denominators linking all four processes. In the context of KOA, age-related decline in antioxidant capacity, combined with chronic mechanical stress and low-grade inflammation, creates a permissive environment for the simultaneous activation of multiple cell death pathways. Notably, the convergence of cuproptosis and ferroptosis on GSH metabolism suggests that strategies aimed at restoring GSH homeostasis—whether through nutritional supplementation (NAC), pharmacological activation of SLC7A11, or inhibition of GSH-consuming enzymes—may offer broad-spectrum chondroprotection. This is particularly relevant given that both copper and iron overload conditions are frequently observed in aging populations and are exacerbated by the inflammatory milieu characteristic of OA.

Another important consideration is the context-dependent role of autophagy. In early KOA, autophagy acts as a critical homeostatic mechanism that mitigates oxidative stress and removes damaged mitochondria. However, as the disease progresses, autophagic flux often becomes impaired or maladaptive, shifting from a protective to a deleterious function. This duality presents both a therapeutic opportunity and a challenge: interventions that enhance autophagy may be beneficial in early-stage disease, whereas late-stage patients may require strategies that normalize excessive autophagic activity. The lack of reliable biomarkers to distinguish these phases remains a significant barrier to precision implementation.

From a therapeutic perspective, multi-target approaches are gaining traction. Preclinical evidence supports the potential of combining copper chelators with iron chelators, or autophagy inducers with apoptosis inhibitors, to achieve synergistic effects. The emergence of nanoparticle-based delivery systems further enhances this possibility by enabling joint-specific targeting, sustained release, and co-delivery of multiple therapeutic agents. Notably, some natural compounds—such as quercetin, curcumin, and resveratrol—exhibit pleiotropic effects that simultaneously modulate several of these pathways, positioning them as promising candidates for further development.

Despite these advances, several critical gaps remain. First, the majority of mechanistic insights are derived from *in vitro* studies using chondrocyte cell lines or primary cells under artificial stress conditions, which may not fully recapitulate the complex *in vivo* environment. Second, rodent models of KOA, while useful, do not perfectly mimic human disease with respect to disease chronicity, joint anatomy, and metabolic context. Third, there is a paucity of longitudinal human data correlating pathway activation with disease progression, treatment response, or clinical outcomes. Fourth, the interplay between these pathways and other disease modifiers—such as mechanical loading, the gut microbiome, and systemic metabolic health—remains underexplored.

Moreover, the translational gap between preclinical promise and clinical application is substantial. Most candidate drugs have poor bioavailability, short intra-articular half-lives, or off-target effects that limit their clinical utility. The development of validated biomarkers for patient stratification is urgently needed to enable personalized therapeutic approaches. Additionally, the stage-specific nature of these pathways suggests that timing of intervention may be as critical as the choice of target.

Looking forward, the integration of multi-omics technologies—including single-cell transcriptomics, proteomics, and metabolomics—will be essential to map the dynamic interactions among these pathways across disease stages. Such approaches may identify novel crosstalk nodes and biomarker candidates that can guide patient selection and monitor treatment response. Furthermore, the development of joint-targeted nanotherapeutics offers a promising strategy to enhance drug accumulation at the site of pathology while minimizing systemic exposure. Ultimately, successful translation will require a paradigm shift from targeting individual cell death pathways to orchestrating a balanced network that preserves chondrocyte homeostasis and prevents cartilage degeneration.

## 7 Conclusion

In conclusion, cuproptosis, ferroptosis, apoptosis, and autophagy represent interconnected pathological mechanisms that collectively contribute to chondrocyte death and cartilage degradation in knee osteoarthritis. While each pathway operates through distinct molecular machinery, they converge on shared regulatory nodes—particularly glutathione metabolism, reactive oxygen species signaling, and mitochondrial function—that orchestrate the balance between cell survival and death. The crosstalk among these pathways, mediated by GSH depletion, ROS accumulation, and key signaling cascades such as mTOR and NF-κB, creates a complex network that amplifies cartilage destruction in KOA.

The dual role of autophagy, ranging from cytoprotective in early disease to potentially deleterious in advanced stages, underscores the importance of context-specific therapeutic modulation. Emerging evidence highlights the promise of multi-target strategies, including combination therapies that simultaneously inhibit metal-dependent cell death pathways and restore autophagic flux, as well as nanoparticle-based platforms that enable joint-selective delivery. Natural compounds with pleiotropic effects offer attractive candidates for further development given their ability to modulate multiple pathways simultaneously.

The stage-dependent and cell-type-specific nature of these pathways underscores the need for personalized, temporally targeted interventions. Development of validated biomarkers and joint-selective delivery systems is a prerequisite for clinical translation. Future research should prioritize the elucidation of pathway crosstalk mechanisms in physiologically relevant models, the development of reliable biomarkers for patient stratification, and the translation of multi-target interventions into clinical trials. Addressing these priorities will be essential to advance the field from mechanistic understanding toward disease-modifying therapies for the millions of patients affected by this debilitating condition. The integration of advanced omics technologies, joint-targeted drug delivery systems, and stage-specific therapeutic algorithms holds promise for achieving precision management of KOA.
